# Virtual Reality in Pain Rehabilitation for Youth With Chronic Pain: Pilot Feasibility Study

**DOI:** 10.2196/22620

**Published:** 2020-11-23

**Authors:** Anya Griffin, Luke Wilson, Amanda B Feinstein, Adeline Bortz, Marissa S Heirich, Rachel Gilkerson, Jenny FM Wagner, Maria Menendez, Thomas J Caruso, Samuel Rodriguez, Srinivas Naidu, Brenda Golianu, Laura E Simons

**Affiliations:** 1 Department of Anesthesiology, Perioperative, and Pain Medicine Stanford University School of Medicine Stanford, CA United States; 2 Mighty Immersion, Inc. New York, NY United States; 3 PGSP-Stanford University Psy.D. Consortium Stanford, CA United States; 4 Lucile Packard Children’s Hospital Stanford, CA United States

**Keywords:** digital health care, virtual reality, immersive technology, chronic pain management, adolescents

## Abstract

**Background:**

In the field of pain, virtual reality (VR) technology has been increasingly common in the context of procedural pain management. As an interactive technology tool, VR has the potential to be extended beyond acute pain management to chronic pain rehabilitation with a focus on increasing engagement with painful or avoided movements.

**Objective:**

We outline the development and initial implementation of a VR program in pain rehabilitation intervention to enhance function in youth with chronic pain.

**Methods:**

We present the development, acceptability, feasibility, and utility of an innovative VR program (Fruity Feet) for pediatric pain rehabilitation to facilitate increased upper and lower extremity engagement. The development team was an interdisciplinary group of pediatric experts, including physical therapists, occupational therapists, pain psychologists, anesthesiologists, pain researchers, and a VR software developer. We used a 4-phase iterative development process that engaged clinicians, parents, and patients via interviews and standardized questionnaires.

**Results:**

This study included 17 pediatric patients (13 female, 4 male) enrolled in an intensive interdisciplinary pain treatment (IIPT) program, with mean age of 13.24 (range 7-17) years, completing a total of 63 VR sessions. Overall reports of presence were high (mean 28.98; max 40; SD 4.02), suggestive of a high level of immersion. Among those with multisession data (n=8), reports of pain (*P*<.001), fear (*P*=.003), avoidance (*P*=.004), and functional limitations (*P*=.01) significantly decreased. Qualitative analysis revealed (1) a positive experience with VR (eg, enjoyed VR, would like to utilize the VR program again, felt VR was a helpful tool); (2) feeling distracted from pain while engaged in VR; (3) greater perceived mobility; and (4) fewer clinician-observed pain behaviors during VR. Movement data support the targeted impact of the Fruity Feet compared to other available VR programs.

**Conclusions:**

The iterative development process yielded a highly engaging and feasible VR program based on qualitative feedback, questionnaires, and movement data. We discuss next steps for the refinement, implementation, and assessment of impact of VR on chronic pain rehabilitation. VR holds great promise as a tool to facilitate therapeutic gains in chronic pain rehabilitation in a manner that is highly reinforcing and fun.

## Introduction

### Background

Virtual reality (VR) is a newly emerging and promising intervention tool for chronic pain treatment to both distract individuals from their pain and facilitate otherwise painful or feared movements [1,2]. For adults exhibiting a variety of chronic pain conditions—including spine, shoulder, abdominal, hip, musculoskeletal, and neuropathic pain—research has demonstrated the efficacy of VR tools, finding significant reductions in subjective reports of pain both during and after VR sessions [3-7]. VR treatments have also been shown to significantly reduce pain, prompt physiological reactions of relaxation, improve physical functioning, and improve social role functioning for adults with chronic low back pain [8,9]. When compared to telephone and internet-based interventions, VR programs are one of the most effective electronic health care modalities for delivering interventions and potentially reducing pain interference within the context of chronic pain [10]. However, most applications of VR for chronic pain remain focused on distraction and pain alleviation, rather than on functional gains through motor and behavioral physical engagement [11].

Moreover, few studies have applied VR tools for pediatric chronic pain [12]. In a recent review of VR studies in pediatric pain (PubMed 2000-2017), there were only 4 that focused on VR for chronic pain, compared to 94 that focused on VR for coping with medical procedures and acute pain [13]. Each of these 4 preliminary studies found VR treatment to be feasible, safe, and potentially efficacious in children with chronic pain conditions. Two pilot studies conducted by Won and colleagues [14] demonstrated a VR program to be feasible and safe for children with complex regional pain syndrome (CRPS). The pilot studies noted qualitative observations of increased relaxation, minimal complaints of pain, and program engagement during the VR sessions. Another pilot study conducted by Shiri and colleagues [15] implemented a 10-session VR and biofeedback regimen over 3 months in youth with chronic headaches. Patients reported significant decreases in headache severity and improved functional outcomes. Despite sparse research on VR interventions for pediatric chronic pain, new work examining specific design factors of VR programs that can enhance youth experience within medical settings sets the context for the development of creative and well-tolerated programming [16].

Beyond pain treatment, VR programs that target physical rehabilitation in children are emerging. Meyns and colleagues [17] developed a VR program, ICT4Rehab, which uses a Wii Balance Board (Nintendo) as a treatment for children with cerebral palsy following lower extremity surgery in inpatient rehabilitation. The study found that patients in both ICT4Rehab and the control group had improvements in sitting balance, with greater improvements noted in the ICT4Rehab group. In another VR system, patients undergoing ankle rehabilitation respond to various VR simulations through interaction with a “Rutgers Ankle” system applying mechanical force to the ankle [18]. The VR simulations seek to improve range of motion, motor coordination, and broad lower extremity function [18]. Taken together, there are considerable opportunities to develop and implement VR programs that target physical rehabilitation in the treatment of pediatric chronic pain.

### Objectives

The goal of this project was to develop a VR intervention to enhance mobility in the presence of chronic pain. Fruity Feet is a VR program created with input from a multidisciplinary team of pediatric pain rehabilitation clinicians, pediatric pain researchers, and VR technology developers. Fruity Feet responds to the growing need for VR applications that specifically target pediatric chronic pain populations who experience limited mobility due to fear and activity avoidance. In this paper, we first describe the development, acceptability, feasibility, and utility of Fruity Feet using a 4-phase iterative development process to facilitate increased upper and lower extremity engagement. We then discuss next steps for the refinement, implementation, and impact assessment of Fruity Feet.

## Methods

### Participants and Setting

Youth admitted to an intensive interdisciplinary pain treatment (IIPT) program were specifically targeted as these individuals typically present with fear of movement and significant physical limitations due to pain. IIPT teams include physical therapists (PTs), occupational therapists (OTs), pain psychologists, pain medicine physicians, and pain medicine nurse practitioners [19,20]. Treatment includes aquatic therapy, individual and group PT/OT/psychology therapy, family therapy, caregiver support, and weekly team care conferences. Many patients admitted to IIPT programs rely on assistive devices to ambulate (ie, walkers, wheelchairs), have limited mobility, and struggle with deconditioning. The goal of the Fruity Feet program was to provide an enhanced, immersive IIPT experience that increased engagement with physical exercises and improved functional outcomes within a difficult-to-treat population.

Eligibility criteria for enrollment in the VR in pain rehabilitation pilot feasibility study included the following: (1) English speaking, (2) aged 7-21 with (3) a diagnosis of chronic pain and the need for physical rehabilitation, and (4) on stable medication/therapy for 2 weeks prior to the initial session. Patients were deemed ineligible if they had (1) diagnoses of neurological conditions (seizures, cerebral palsy, developmental delay) or (2) severe psychiatric symptoms (severe depression/anxiety).

### Development and Prototyping

The IIPT pediatric pain rehabilitation team worked collaboratively with the CHARIOT technology software design team and Mighty Immersion, Inc. to develop Fruity Feet, a unique VR experience for youth undergoing chronic pain treatment and rehabilitation. This team incorporated an iterative process to develop this VR intervention, building upon patient and clinician feedback to improve acceptability and utility. The development of Fruity Feet consisted of 4 phases: Needs Assessment, Prototyping, Iteration and Refinement, and Feasibility and Acceptability. The Needs Assessment phase involved gathering information from the IIPT team to determine the scope of clinical needs and establish parameters for the VR intervention, hardware, and space. The Prototyping phase involved designing the initial functionality for lower extremity movement. The Iteration and Refinement phase involved a cyclical process of (1) testing the program with clinicians and test case patients, (2) gathering feedback, and (3) adjusting the software design, space, and hardware needs accordingly.

**Figure 1:**
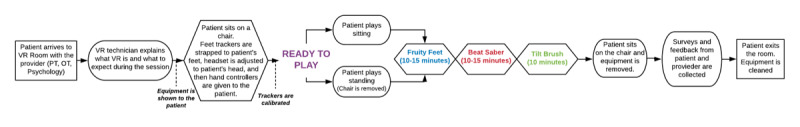
VR session flowchart. Patient arrives to session with clinician who orients patient to the use of VR equipment safely. While seated, foot and hand trackers are placed, and VR headset adjusted on patient. Clinician has patient begin while seated initially to orient to VR system and then eventually standing, as deemed appropriate. Three VR programs were utilized for approximately 10 minutes each (Fruity Feet, Beat Saber, and Tilt Brush). After the VR session, patient once again is seated to remove trackers and headset. Patient completes survey and both patient and clinician give feedback after each session. Equipment is thoroughly cleaned and sanitized following each session. VR: virtual reality.

The Feasibility and Acceptability phase ran in parallel to the Iteration and Refinement phase. Following Institutional Review Board approval, eligible patients that were enrolled in the IIPT at Stanford Children’s Health were invited to participate in feasibility testing. After consent from parent and assent from child were obtained, VR sessions were incorporated into the patient’s IIPT schedule. VR sessions occurred once weekly for approximately 30 minutes. Figure 1 describes a typical VR session. Parents were not present during VR sessions, but were given the opportunity to observe the session either live behind a one-way mirror or via recorded video footage after the session. After the session, the child, parent, and clinician present completed measures or interview questions. A technician was also present to manage the session flow and administer the measures and questions at the end. The primary developer (LW) also attended multiple sessions to inform the iterative development of Fruity Feet.

### Outcome Measures

After each session, patients were asked to complete the following questionnaires related to their experience while in VR.

#### Presence Questionnaire

Patients were asked to provide a rating, ranging from 0 (not at all) to 4 (very strongly) to 10 questions, assessing the patient’s perception of how real the virtual world seemed and whether they felt their virtual body (avatar) was an extension of their own. Higher scores are suggestive of greater presence in the virtual environment [21].

#### Child Daily Questionnaire

The Child Daily Questionnaire was developed for repeated administration in the context of pediatric pain clinical trials [22]. The Child Daily Questionnaire consists of 13 items assessing pain and functioning in the last 24 hours. Eleven of the daily items were pain related: worry/fear (2 items), avoidance (2 items), functional limitations (3 items), activity engagement/acceptance (2 items), and reactivity (2 items). These items and domains are derived from validated full-version measures. These 11 items are rated on a 100-point visual analog scale ranging from strongly disagree to strongly agree. Item 12 assesses current pain (ie, pain felt in the last 24 hours) on a numeric rating scale from 0 (no pain) to 10 (worst possible pain). Item 13 includes an open text box for the patient to describe anything exciting or stressful from the past 24 hours.

After each VR session, patients, parents, and clinicians were also asked to provide feedback in an open-ended interview format (Textbox 1).

### Statistical Analysis

Descriptive statistics for all questionnaires were run and repeated measures mixed models were run for the multisession Child Daily Questionnaire data using SPSS 25 (IBM). For interview data, NVivo qualitative statistical software was utilized to analyze participant (patient, clinician, parent) responses provided following VR sessions. Interview responses were imported into NVivo and case nodes were set up for each participant. The material was explored and emerging themes were coded. Text was searched and word frequency queries were placed into those coded themes. Themes from interview feedback responses with the most frequency were summarized into visual charts for patients and clinicians.

**Post-VR interview questions. VR: virtual reality.**

**Patient questions**

- What was it like to be in VR?
- How did it feel to be in VR?
- Tell me about any of the feelings you are experiencing now after being in VR.
- Tell me about what happened when you were in VR. Tell me about the parts that you expected? Tell me about the parts that you did NOT expect?
- Now that you have tried VR, how do you think VR can help other kids?
- How do you think we could improve the VR experience for other kids?
- If you could change anything about this, how would you make it better?
- Questions about the process of learning to use the avatar: eg, “How did you learn to use the VR character?” “Which bits were easy to learn?” “Which bits were hard to learn?”
- Feelings of ownership and control of the avatar: eg, “How did you feel you could control your virtual character?” “How was it difficult to control? How was it easy to control?”
- Feelings of presence or immersion: eg, “How ‘real’ did it feel to you?” “When you were in the virtual world, what did you notice was happening around you in the real world?” “What else did you think about?” “Where did you have your attention focused?”
- What they consider to be the biggest benefits and failures of VR therapy eg, “What are the best things about the virtual reality?” “What are the things you don’t like about it?” “What would you do differently?”
- Would they consider doing VR therapy often as a more intensive treatment program? eg, “How often would you be willing to come back to the clinic to do more VR?” “How much do you think doing VR regularly would help with your pain?” “How would you compare VR to other treatments for pain?”

**Parent questions**

- What do you think or how do you feel about your child’s VR session today?
- Is there anything that could be improved in the VR sessions?

**Clinician questions**

- How did the VR session go today?
- Is there anything that could be improved in the VR sessions?

## Results

### Participants and Setting

**Figure 2:**
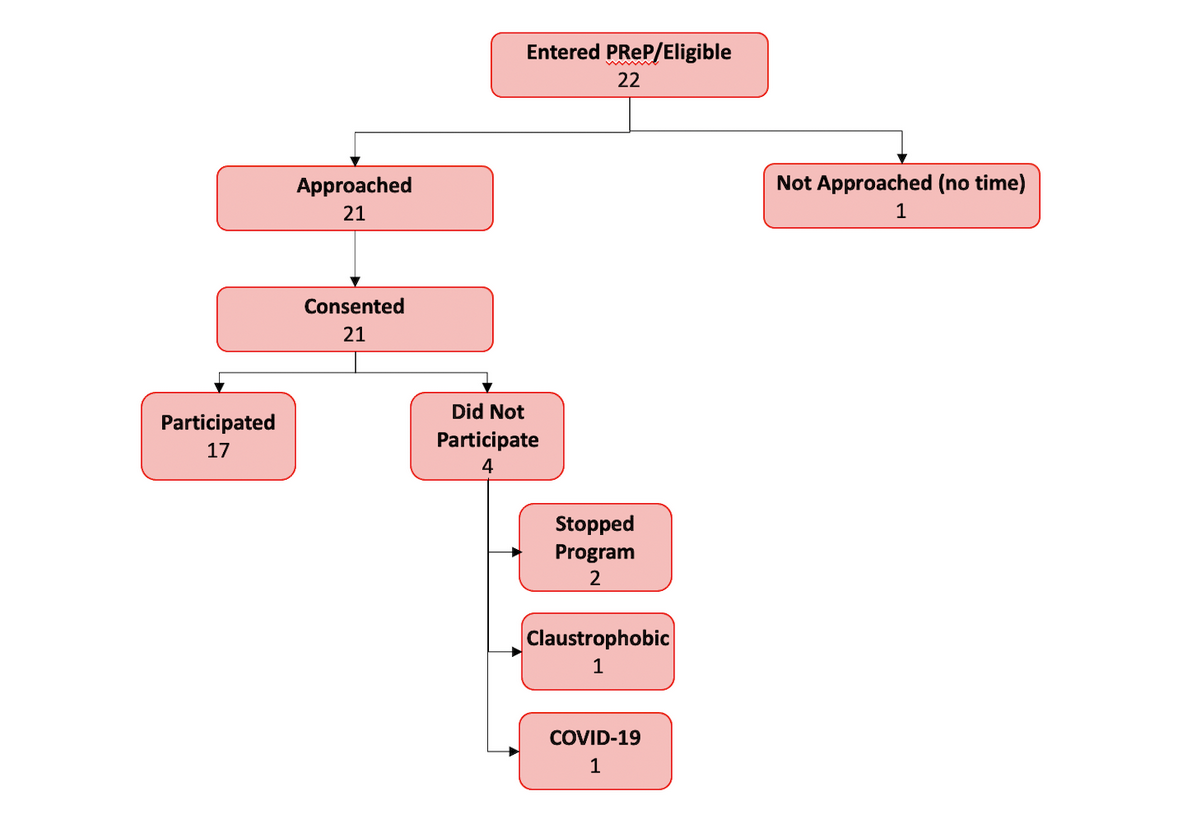
Virtual reality CONSORT flowchart.

Seventeen, predominantly female (13 female, 4 male) youth with a mean age of 13.24 (ranging from 7 to 17 years) being treated in an IIPT from January 2019 to March 2020 participated in this feasibility pilot (Figure 2). Patients had a variety of chronic pain conditions (ie, lower extremity CRPS=9, primary musculoskeletal pain [diffuse/widespread and localized]=6, Ehlers–Danlos syndrome=1, irritable bowel syndrome=1). Patients presented having experienced pain for an average of 17 months (range 1-60 months). Patients were in IIPT treatment for an average of 7 weeks (range 4-12 weeks). Patients participated in an average of 4 VR sessions (mean 3.71 sessions), with a range of 1-8 total sessions. For all but 1 patient, the number of sessions was contingent on availability of VR technician and duration of IIPT admission. One patient withdrew after her third VR session due to dizziness and photophobia. No other adverse events were reported.

Across 17 patients, 63 VR sessions were completed. All sessions were conducted with a clinician (ie, PT, OT, or pain psychologist) present in a dedicated VR room with flooring for physical activity use, such as floor mats typically used in a rehabilitation gym for safety. VR sessions were typically 30 minutes in duration (mean 29.57; median 30.08; range 6.16 to 80.1). The clinician determined the appropriate treatment and chose the duration as well as the mode that was needed for each patient while engaged in VR. Clinicians ensured safety parameters within the space and considering patient ability and function.

### Development and Prototyping

The first phase of the project was dedicated to program start-up, which included identifying needs for the VR intervention, obtaining Institutional Review Board approval, outfitting a permanent testing room for VR use, and training clinicians on how to administer a VR session. The team initially met to establish the parameters for the intervention, following an extensive exploration of existing VR programs for rehabilitation purposes. The team found that existing rehabilitation programs focused solely on upper extremity involvement. The team deemed these existing programs inadequate for the pediatric pain rehabilitation population, which presents with a greater need for lower extremity interventions within a more age-appropriate and engaging platform. The needs assessments with pediatric clinicians (ie, physical therapy, occupational therapy, pain psychology, and pain medicine physicians) further defined the goals and recommendations for VR interventions in pediatric pain rehabilitation. PTs requested activities targeting functional goals to strengthen muscles and increase range of motion. OTs requested movements targeting activities of daily living. Pain psychologists provided insights into fears about pain related to movement during OT and PT sessions. Pain psychologists also requested options for modifying the delivery format for youth who may be distressed by wearing a headset or too anxious to begin lower extremity VR in a standing position. Pain medicine physicians requested options for patients who were sitting, standing, or otherwise limited in their physical abilities.

Following clinician needs assessments, the software design team shadowed each clinician to observe youth with chronic pain during typical IIPT sessions. This process provided the software design team with use cases and insight into the tasks youth were attempting to master in physical therapy, occupational therapy, and pain psychology sessions (eg, functional goals for lower and upper extremities, methods to increase mobility, use of pain coping skills to manage pain symptoms during treatment, the benefits of distraction from pain). Understanding these tasks, as well as the process and protocols within each therapy session allowed the software design team to further define an optimal VR tool for IIPT—an immersive VR world that would promote engagement in gamified pain rehabilitation tasks that scale to meet treatment demands based on a patient’s current ability.

Following the clinician needs assessment and initial program software design, the technical team was able make decisions about the VR hardware and room setup. The team chose to use an HTC VIVE VR System, which tracks a user’s head in a 10′ × 10′ play space with 6 degrees of freedom. The VIVE VR System also includes 4 additional 6 degrees of freedom trackers, which track the position and rotation of the player’s hands and feet. Using this system, users can fully immerse themselves in a virtual world, with the ability to walk around and touch virtual objects with their hands and feet. The VIVE VR System requires a VR-ready computer and 2 external trackers (lighthouses) positioned in opposite corners of the play area. In order to house this system, the program used a dedicated VR room with adequate space for the hardware setup, which included a 10′ × 10′ play area, a locked cabinet for storing the computer and other VR equipment, 2 permanently mounted VR lighthouses for quick session setup, a retractable cable management system to help keep the VR headset’s cable out of the way during a session, and rubber gym flooring for safe, active VR usage.

The software design team began by focusing on a single game, Fruity Feet, to prototype and test with patients. Fruity Feet gamifies lower extremity PT, helping patients increase their range of motion and become more comfortable with moving their feet and legs. Gameplay mechanics were built around the following lower extremity PT movement goals: multiplanar stepping (ie, forward, side, back), stomping, marching, kicking, raising leg to different heights, and active ankle range of motion tasks (ie, dorsiflexion, plantarflexion, inversion, and eversion). Importantly, the module was also built to scale to a patient’s mobility, ensuring that patients of all abilities could play the game and benefit from the VR intervention.

**Figure 3:**
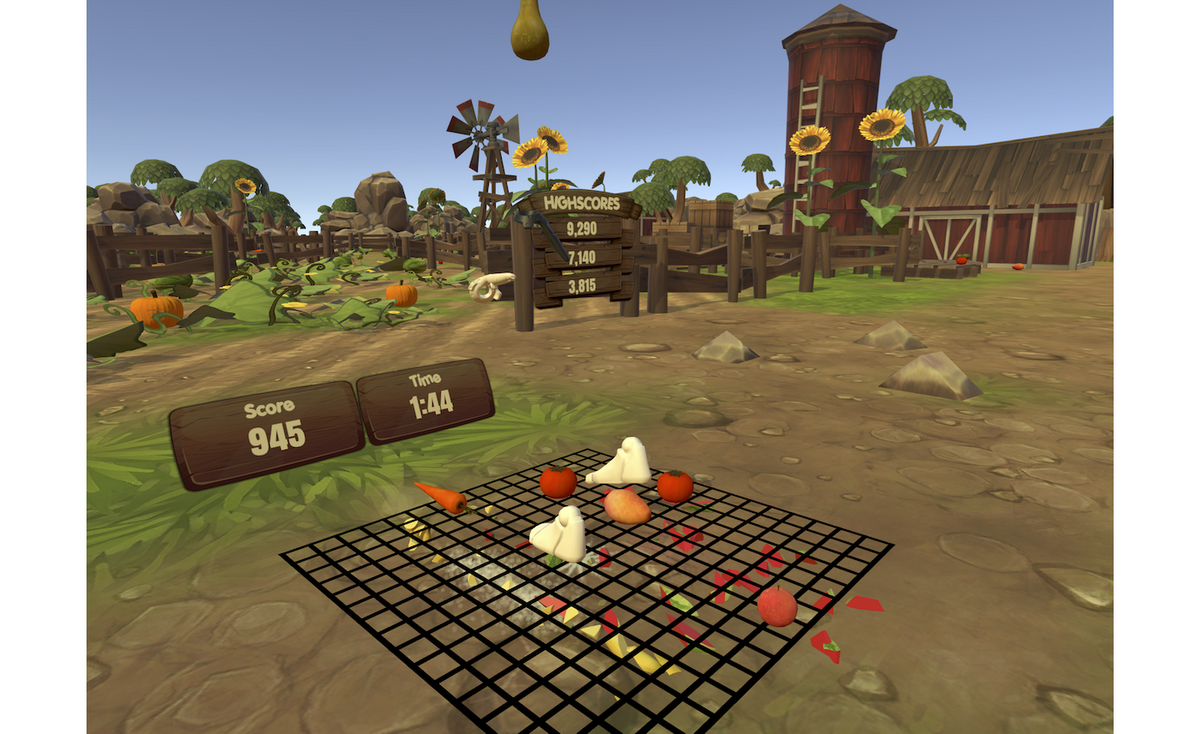
Fruity Feet Gameplay. The player embodies the avatar feet and hands, using them to squish virtual fruit. The player must stomp on as much fruit as possible before the timer runs out. Players are awarded points based on how quickly and effectively they stomp on the fruit. Their score is tracked in real-time and they can keep track of previous high scores to try and beat their old records. The virtual world is built to look as if players are on a farm to further immersion and provide an engaging game environment.

**Figure 4:**
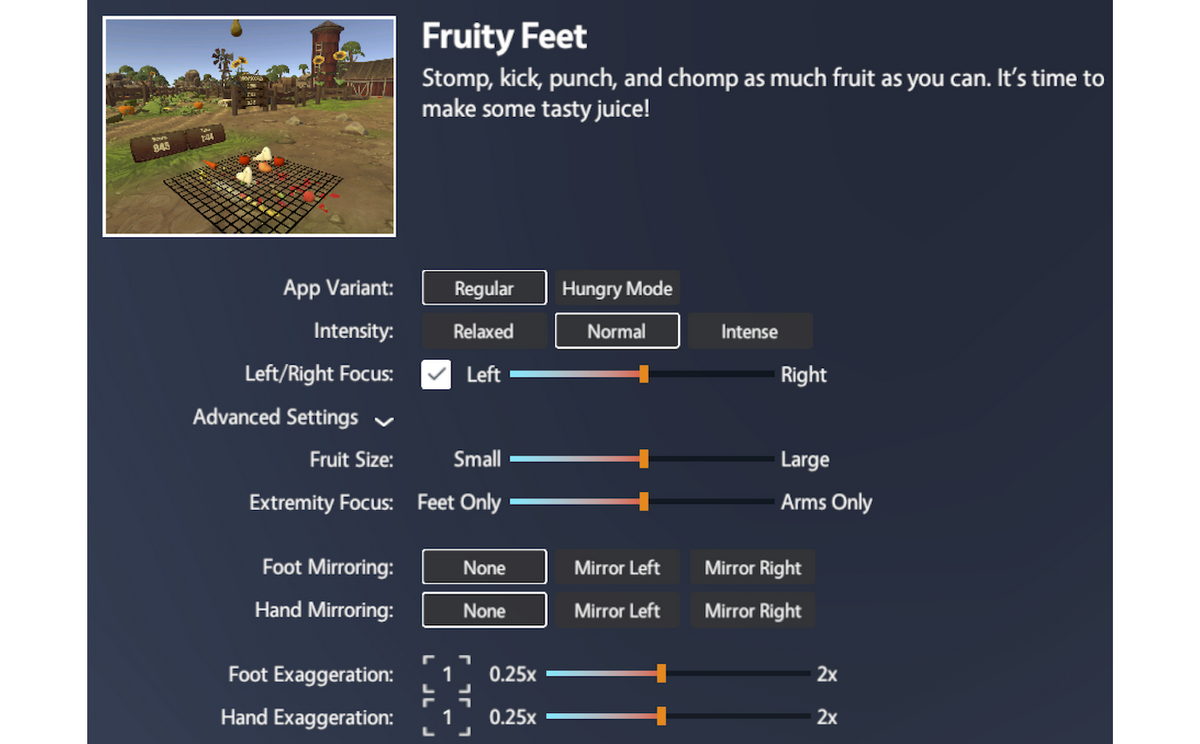
Fruity Feet control panel. Using the control panel, clinicians can adjust the game to better fit the needs of their patient. Intensity affects the rate at which fruit appears in the world. Left/Right Focus focuses the game activity on the left/right side of the patient, encouraging patients to use their affected side. Fruit Size changes how high players must lift their foot in order to effectively stomp a fruit. Extremity Focus focuses the game activity on the player’s lower or upper extremities. Foot/Hand Mirroring enables an experimental mode that mirrors the virtual extremity, much like mirror therapy. Foot/Hand Exaggeration enables an experimental mode that affects the movement gain of the virtual avatar's feet and hands (higher exaggeration results in the virtual avatar moving farther than the patient moved in the real world, and lower exaggeration results in the virtual avatar moving less than the patient moved in the real world).

The team designed Fruity Feet to be developmentally appropriate for children and adolescents. The game is fun, light-hearted, and often silly, while employing stylized graphics, in-game feedback, and sound design to offer immersive and engaging game play. During play, users are placed on a farm and instructed to stomp and kick as many virtual fruits and vegetables as possible in order make juice and gain points before a timer runs out. Player step/stomp quality is measured by the VIVE sensors, scoring players based on how high they raise their legs and whether they land their feet in the center of the fruit. As players stomp and kick at the fruit appearing beneath their feet, virtual fruit juice splatters the world and a cartoon farmer yells encouraging phrases such as “Nice job!” and “That’s some juicy fruit!” (Figure 3). Clinicians can adjust aspects of the game to tailor the VR experience to a patient’s ability level and other needs using the game’s control panel (Figure 4).

Following the initial development of Fruity Feet, the team began a cyclical process of testing the program with clinicians/patients, gathering feedback, and iterating on the software design. This feedback loop was critical in building a VR module that was easy to use, fun to play, and met the appropriate therapy goals for patients and clinicians. During this process, both Fruity Feet and commercially available VR programs were used to obtain feedback to further develop Fruity Feet and the VR environment. Providers noted the importance of including both upper and lower extremity engagement during Fruity Feet tasks. For example, an OT on the team provided feedback about neck discomfort while constantly looking downward when the task was initially solely focused on lower extremity movements. One of the first patient test cases initially presented with mobility limitations, using one crutch to ambulate. The patient was a 12-year-old Caucasian female with a diagnosis of CRPS of the lower right extremity. She struggled with tasks such as standing, walking, and other movements that required her to bear weight on her right foot. She regularly participated in VR sessions during her rehabilitation process, and as her pain and function began improving, she requested more challenges and further gamification of Fruity Feet. As an avid gamer herself, she requested a competitive element for the game, and even went so far as to suggest an “alien invasion” component when players got to a certain level. With this patient’s feedback, the team developed a new game add-on in which unknown flying objects (UFOs) appeared, challenging advanced users with faster-flying fruits.

Fruity Feet was eventually expanded to include new game modes. These modes were designed as a modification to allow the use of VR for lower extremities from a seated position. This prompted the development of a graded process for increasing lower extremity active range of motion and weight bearing by transitioning from seated tasks (ie, active multiplanar ankle movements, active knee flexion and extension, and multiplanar weight shifting for increased weight bearing) to standing tasks (ie, supported standing, weight shifting, squatting, single limb weight bearing, and balancing). Game modes also included the use of upper extremities, creating an active and dynamic experience for patients pursuing both upper and lower extremity goals (ie, reaching, throwing, hitting, kicking, stomping, squatting, standing, twisting, and balancing), all at once. Beyond specific movement goals, these new game modes provided variety and choice for patients using VR, which made for a more engaging and less repetitive session.

Modifications helped to simulate activities of daily living, play, and leisure activities while downregulating the sensory system. The movements within Fruity Feet helped to promote increased function for daily living tasks. Fruity Feet engaged participants in leisure activity that required movement, balance, and endurance, which could be generalized to age-appropriate occupations including the act of raising one’s arms above the head in order to don a shirt (eg, the affordance of the sling shot task), weight shifting, and standing balance skills necessary for showering (eg, stomping on virtual fruit of various sizes and locations while standing) and functional endurance activities important for school, sports, and other active recreational tasks (eg, gamification increased length of time engaged in the VR tasks). Each change and addition to Fruity Feet went through multiple iterations and feedback sessions with clinicians and patients. As the tasks became more dynamic, clinicians continued to identify and ensure safety measures were considered through each iteration of Fruity Feet (ie, need to monitor patient’s movements while in VR, need for a visual barrier within VR). Many patients in the IIPT program were able to see their suggestions and feedback come to life in VR as requests for additions, such as farm animals and aliens, were incorporated into the software.

One example of how Fruity Feet was improved during the iteration and refinement phase for wheelchair-bound patients involves a 12-year-old Caucasian female patient who was nonweight bearing yet eager to participate in the VR in pain rehabilitation platform. The patient was diagnosed with CRPS in both of her lower extremities. She initially struggled with tasks of standing or stepping but seemed to benefit from active ankle range of motion tasks conducted while seated in a chair. This patient provided feedback to include rewards in addition to points, such as being able to add animals to the virtual farm in exchange for a certain amount of points earned. This addition improved motivation to remain engaged in the game for longer periods and allowed the patient to progress to more dynamic standing and stepping goals. The patient struggled to achieve these movement goals during her typical PT sessions in the IIPT program without VR in pain rehabilitation; however, once VR was added, she was able to attain such goals more effectively. The patient was able to increase use of her lower extremities, resulting in an improved ability to ambulate without assistive devices. She noted that Fruity Feet improved her motivation to engage in standing/walking IIPT goals with the PT.

### Outcome Measures

#### Presence and Daily Questionnaire

**Figure 5:**
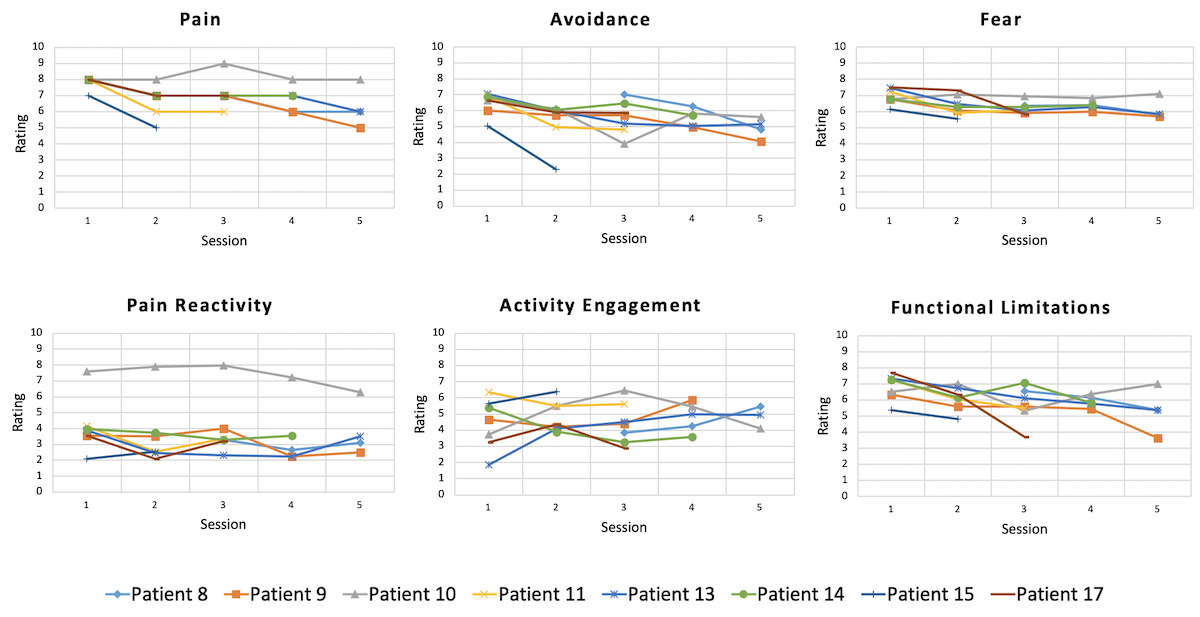
Multisession ratings of pain, fear, avoidance, activity engagement, and pain reactivity. Each line represents a patient’s multisession post-VR Child Daily Questionnaire ratings. As can be observed from the graphs, number of sessions/ratings ranged from 2 to 5. Repeated measures mixed model analyses revealed significant effects for time with decreases in pain, fear, avoidance, and functional limitations. No effects for time were observed for activity engagement or pain reactivity. VR: virtual reality.

After each VR session, patients were surveyed on their perception of the virtual character (avatar) as well as the virtual environment. Patients were asked about how real the virtual world seemed (eg, immersion), and whether they felt the avatar’s body was an extension of their own body (eg, embodiment). Overall reports of presence were high (n=17; mean 28.98 [SD 4.02]), with scores ranging from 0 to 40, with higher scores suggestive of greater presence. As the child daily questionnaire began after the feasibility pilot started, we provide data on those who completed it more than once (n=8; Figure 5). The repeated measures mixed models showed a significant effect for time with decreases in pain (*F*_4,27.7_=9.27, *P*<.001), fear (*F*_4,25.7_=5.17, *P*=.003), avoidance (*F*_4,27.9_=4.96, *P*=.004), and functional limitations (*F*_4,25.2_=4.20, *P*=.01) across VR sessions. Activity engagement and pain reactivity reports did not significantly change across VR sessions.

#### Child Interviews

There were a total of 63 child interview responses from 17 patients completed after VR sessions. Feedback was obtained for the first 45 sessions (n=13), as part of the iterative process, following each VR session. These 45 responses were entered into the NVivo qualitative analysis software system. An emergent coding approach was utilized, with 2 coders (AG and AF) creating the same themes, to demonstrate accuracy, as responses could be coded in various ways. Multiple responses informed the 4 derived themes with sample responses detailed in Table 1.

**Table 1:** Sample responses from participants across the 4 themes.

Participant ID	Positive response	Distraction from pain	Increased function	Decreased pain
6	*I would be willing to come to do VR for sure. It is like a more fun version of PT.* [Session 3]	*The biggest benefit is being able to do motions I wouldn’t normally be able to do without thinking of the pain.* [Session 3]	*I could move my body and balance on my bad leg.* [Session 3]	*It was very distracting. It made it a lot easier to move my leg without pain.* [Session 3]
8	*Reaching my standing goals in an easy way and the time goes faster.* [Session 4]	*It distracts me from pain.* [Session 5]	*I can put more weight on my foot.* [Session 5]	*It can help you to forget about your pain.* [Session 4]
11	*VR keeps you busy and distracted from the real world, the time flies and I can be standing easily for a long time.* [Session 1]	*It can distract you and you are able to move more.* [Session 1]	*Standing for 40 minutes with no breaks and I can walk around the room for a long period of time.* [Session 1]	*I forgot about my pain.* [Session 1]

These emergent patient response themes included (1) positive experience or response to the VR session (eg, enjoyed experience, would like to utilize this VR program again, felt this was a helpful tool; 40/45 responses, 89%); (2) feeling distracted from pain while engaged in the VR task (eg, distracted from pain symptoms during VR tasks; 24/45 responses, 53%); (3) VR increased physical function/mobility (eg, achieved functional goal, able to complete more physical tasks such as standing/walking/stomping; 16/45 responses, 36%); and (4) reduced pain behaviors/symptoms (eg, decreased pain level, noted feeling painless) during VR (10/45 responses, 22%).

We highlight responses from 1 participant who demonstrated progress over 3 weekly sessions. This patient identified initially not liking the immersive experience, but agreed to continue exploring the VR platform. Over the subsequent 2 sessions, the patient’s responses improved to conclude that Fruity Feet was acceptable and noted improvements in function without noticing pain with movement (Table 2). The patient also recommended this intervention for other youth with chronic pain.

**Table 2:** Interview responses from one patient across multiple VR^a^ sessions.

Session	What was it like to be in this VR?	How did it feel to be in this VR?	Do you think VR can help other kids with chronic pain?	What are the things you like/don’t like about this VR?	How often would you be willing to do more VR?
1	*Weird*	*I felt trapped. I felt I couldn’t control what I was doing.*	*May be*	*I don’t like to be so immersive*	*Maybe in the future*
2	*Today I liked it. It was good*	*I felt better than the last time. I could control the virtual world*	*It can help kids to stand more time and use their legs more without noticing discomfort*	*You can forget a little bit about your pain.*	*Yes, I will try it again.*
3	*Today I loved it. It was easy to reach my standing goal*	*It was good*	*Sure, VR can help me and other kids*	*I can be standing more time without any pain*	*Once a week is good for me*

^a^VR: virtual reality.

Most patients reported a positive response to the VR experience. Patients reported that it felt real, and that they felt immersed in the VR world, even while speaking with the clinician in the room during the session. Nearly all patients, in at least one of their sessions, reported feeling distracted from their pain:

Painless. It's ... kind of like a coping thing where you're distracted and ... you don’t feel like you have pain. You’re just trying to focus on whatever game you're doing

ID 1, Session 1

It was really neat! It made me forget about the pain a little because I was in a world where the pain didn’t exist

ID 6, Sessions 1 and 2

Patients also reported experiencing ownership and control over their avatar during the VR sessions, and for some this increased over subsequent sessions. All patients responded in at least one session that VR would help other youth with chronic pain. All patients responded that they would use VR in the IIPT program, with several commenting that it was more “fun” or “distracting” than alternatives such as physical therapy sessions without VR. Many patients also responded with increased functional gains during the VR task, reporting:

When I was in VR I was able to move my foot around much more in order to squash all of the fruit

ID 6, Session 1

#### Clinician Interviews

An emergent coding approach in NVivo was also utilized with clinician responses, with 2 coders (AG and AF) creating the same themes, demonstrating accuracy. Qualitative results generated frequency scores, resulting in 4 primary themes for clinicians derived from 32 responses from 5 clinicians (1 PT, 1 OT, and 3 pain psychology). Clinician response themes about observations of their patients included the following: (1) increased function/mobility (N=17 responses; 53%), (2) enjoyment of the VR experience (N=12 responses; 38%), (3) VR extended or lengthened patient’s ability to engage in physical activity (N=9 responses; 28%), and (4) VR increased patient’s distraction from pain symptoms during the VR sessions (N=8 responses; 25%).

Clinician feedback suggested that VR helped their patients achieve pain rehabilitation goals. Some credited VR with helping to progress patients to no longer need assistive devices.

He really comes to life when he's doing VR. So, it's nice to see him so active, animated ... the point where he's singing and he's dancing, and he's really blossomed with what he can do with the game

ID 1, Session 4

She didn't even mention a concern about the fact that she was standing for nearly 40 minutes ... didn't complain, didn't say anything, and she was shifting her weight onto the affected limb ... standing equally or even on the right leg, pretty frequently

ID 2, Session 5

Some clinicians commented that VR was a useful environment for overcoming psychological barriers.

It's kind of like nice to be able to tie in some of the things we're working with in psychology, with what he was doing in VR ... and (it) was sort of the perfect kind of concrete example of being able to put that into action

ID 1, Session 5

#### Parent Interviews

When available, parents were interviewed about their child’s VR experience. Derived from 4 responses from 3 parents, parents remarked on the immersive quality of VR at their child’s level of engagement while playing the games.

I love it... And the most impressive thing was that she can walk around without help and without complaining about her pain.

ID 8, Session 1

It gets her moving a lot more than she thinks she can

ID 2, Session 7

One parent suggested that it would benefit their child to watch footage of their own motion after the session, to “change her mind” about how much she can do [ID 2, Session 7].

### Areas for Improvement and Modification

Based on clinician observation and interviews, areas for improvement or modifications were identified to attenuate any adverse effects from VR engagement. For example, there was 1 patient (ID 5) that reported feeling “trapped” and “out of control” during her first session, which was discontinued. Rather than engaging in VR with the headset, a projector was utilized to illuminate the gaming images on the wall, because the patient was interested in finding a way to comfortably participate in the VR intervention. She returned for additional sessions with this modification, and during her third session, the patient commented that she “loved it.” The patient and clinician both suggested that more games be made available in projector mode for patients who do not feel comfortable with the immersive VR experience. Furthermore, 2 patients reported feeling “dizzy” or “weird” after sessions, with 1 patient noting that it was difficult to become accustomed to light and other sensory stimuli in VR (ID 10, Session 3). This patient later reported having a headache prior to participating in the session, which may have been exacerbated by the light in VR. One patient reported that the headset was heavy (ID 9, Session 1).

### Comparing Fruity Feet With Commercially Available VR Programs

**Figure 6:**
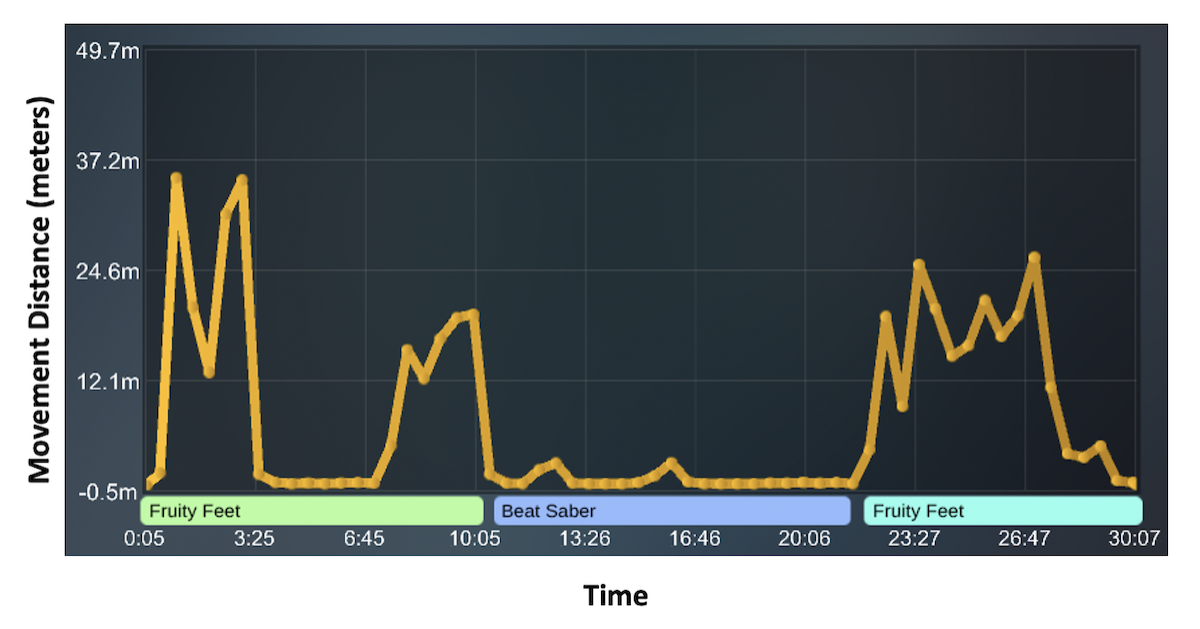
VR Clinical Comparison Research Tool. The screen provides a graph of the patient’s progress over time during their VR session while using Fruity Feet and a commercially used program (eg, Beat Saber) and then utilizing Fruity Feet once again. The graph shows increased movement of the lower left extremity (yellow line) while engaged in Fruity Feet. The lower left extremity movement reduces with other VR programming (eg, Beat Saber). VR: virtual reality.

Clinically, clinicians noted that patients moved their lower extremities more frequently with Fruity Feet in comparison to other VR programs. The VR team then compared Fruity Feet to other commercially available VR games and programs (eg, Beat Saber by Beat Games, Tilt Brush by Google). In order to capture differences in movement across these programs, the software developers created a novel method to compare movement while using different VR programs called the VR Clinical Comparison Research Tool. The VR Clinical Comparison Research Tool tracks the movements of each extremity simultaneously in real time with advanced analytic capabilities for range of motion across VR programs. Clinicians can create custom play lists, leveraging existing content with custom modules, while tracking progress within each session and longitudinally throughout the course of therapy. This allows for comparison across VR programs for VR in pain rehabilitation. Preliminary data for 1 sample case demonstrated that Fruity Feet tracked increased movement for a patient with CRPS in the patient’s affected left lower extremity (Figure 6).

## Discussion

### Principal Findings

This paper describes the development and implementation of a novel VR intervention for youth with chronic pain participating in an intensive IIPT program. Existing game-based VR programs as well as rehabilitation-centered VR programs do not currently meet the physical rehabilitative needs of pediatric patients with pain, particularly due to the preponderance of those suffering from lower extremity pain and mobility limitations. To address this opportunity for meaningful VR engagement in rehabilitation, we implemented a multistep and iterative process of treatment development with a cohort of individuals admitted to an IIPT. The intervention described in this paper, Fruity Feet, was born out of a collaboration between pediatric pain researchers, clinicians on the front line with patients (physicians, PTs, OTs, and pain psychologists), and software developers interested in designing a VR program specifically to meet the needs of pediatric patients with chronic pain participating in functional rehabilitation. The result is a gamified VR program that has unique lower extremity capabilities and that is adaptable to each individual’s mobility.

To develop Fruity Feet, a needs assessment with rehabilitation clinicians was critical to understand the unique ways that youth struggle to engage in therapies, and potential ways in which VR could not only enhance their participation in therapy, but also benefit their progress. Furthermore, understanding specific movements that are often avoided due to pain and fear provided targets for Fruity Feet optimization (eg, isolated ankle movements). After the prototyping phase, patient involvement was critical to the iteration and refinement of the Fruity Feet game developed. In this initial phase, feasibility was high, particularly with a captive audience of youth participating in an IIPT 5 days per week with multiple therapy sessions per day. Patients often preferred and even requested that their therapy session include VR, demonstrating high acceptability as well. Qualitative data from patients and parents reflected engagement in activities not previously perceived possible (eg, extended duration, increased mobility), distraction produced by VR, and therapies with VR described at times as “fun.”

The Fruity Feet program yielded results consistent with prior VR therapies in the treatment of pediatric pain, as it was both tolerable and safe [14]. Moreover, subjective clinician, parent, and patient report of engagement, distraction, immersion, and enjoyment of the process were consistent with prior work [14,23,24]. The multisession daily reports indicated decreased pain, fear, avoidance, and functional impairments across VR sessions and this result is consistent with prior work [15,25-27]. Given that these changes were observed in the context of IIPT, it is possible the changes reflect general versus VR-specific effects, and thus it is necessary to conduct a more controlled pilot trial to measure the impact of VR on pain rehabilitation. Lastly, in alignment with a holistic model for VR program design developed by Ahmadpour and colleagues [28] in which autonomy, control, and empowerment are outlined as important design considerations, Fruity Feet yielded qualitative patient feedback of ownership and control over one’s avatar during sessions.

### Limitations

There are several limitations with regard to the implementation and generalizability of our VR program. Equipment setup for sessions using VR was typically more time-consuming than typical PT or OT sessions. A dedicated VR room with specifications for optimizing safety (eg, 10′ × 10′ play area, rubber gym flooring, mounted VR lighthouses, cable management system) was needed for administering sessions due to space required for conducting the physically active VR sessions. Although technical issues were uncommon, our clinicians did have a dedicated technology expert available for assistance during all sessions. Expense of equipment, staffing, and space constraints may limit adoptability by other institutions. The clinical team also reported difficulty with patients generalizing their progress outside of VR sessions at times (eg, standing for 40 minutes in VR session, whereas only being able to stand for 10-20 minutes in a subsequent PT session). This suggests the importance of potentially implementing VR in pain rehabilitation over the course of several sessions to examine the cumulative impact of VR on skill generalization. In addition, it was not possible for patients to utilize the VR equipment outside of session which limited their abilities to emulate their therapy sessions when completing their home exercises. Interestingly, some families with resources to purchase VR headsets and gaming consoles requested guidance on purchasing their own; however, Fruity Feet is not yet commercially available, limiting their ability to continue with the therapy-inspired modules after participation in the IIPT. A further limitation and caution arises in terms of sanitary reuse of equipment. Although our team disinfected the headsets and controllers prior to each patient’s participation, additional health and safety concerns must now be considered in a time even more focused on infectious disease prevention. Consideration should be made for also utilizing a UV sanitizing device along with the standard disinfecting process. Lastly, applying a single-case experimental design approach in future studies would allow for a more sensitive analysis of change processes as the current data collected limited the conclusions we could draw about the impact of VR in pain rehabilitation on functional outcomes.

### Future Directions

Future developments of VR in this context will focus on testing with additional samples, as well as further testing of *mirror therapy* and *exaggerated movement* modules. In addition, a clinical protocol for the *exposure therapy* and *range of motion* exercises is underway. We have continued to develop tools for pain psychology interventions with the addition of a heart rate variability biofeedback component connected to VR (currently in the prototype phase). This tool will include the use of pain management coping skills with the benefits of heart rate variability to explore an immersive and interactive VR task, while fostering relaxation and self-regulation for downregulation of the nervous system. Furthermore, we have also been testing the use of VR with pedaling for increasing a variety of seated tasks. It will be critical to also assess sustained benefits from VR after active treatment across domains of function. Finally, we have begun expansion of VR in pain rehabilitation to multiple sites in the United States and Canada to examine the feasibility of dissemination and the effectiveness of this VR intervention across a large, diverse population of youth with chronic pain.

### Conclusions

The VR application Fruity Feet has the potential to make a tremendous impact on the rehabilitative treatment of youth with chronic pain. The iterative process has helped to improve and refine this resource, with customized settings for a specific extremity, mirror therapy, exaggerated movement capabilities, and a VR Clinical Comparison Research Tool that tracks the movements of each extremity in real-time with advanced analytic capabilities across VR programs. Preliminary data suggest improvements in movement with decreased focus on pain symptoms while immersed in the VR world. VR in pain rehabilitation helped youth with chronic pain in an IIPT program to increase distraction from pain and helped to improve function to achieve rehabilitation goals. Furthermore, VR in pain rehabilitation successfully incorporated the use of lower extremities, in addition to upper extremities, which allowed both sitting and standing tasks for improved patient accessibility and generalizability. Youth with chronic pain found VR in pain rehabilitation to be acceptable, feasible, and engaging.

## Abbreviations

IIPT: interdisciplinary pain treatment
VR: virtual reality
